# Style

**Published:** 1882-07

**Authors:** 


**Style.**—Some men of vigorous minds, but more conversant with things than with words, and who, having never studied composition as an art, have not learned that real force of style must be effortless, and consists mainly in its simplicity and appropriateness; they fancy that common words are not half strong enough to say what they want to say; and so they try to strengthen them by writing them in a different character. Men of science do this; for words with them are signs, which must stand out to be conspicuous. Soldiers often do this; for, though a few of them are among the most skillful in the drilling and maneuvering of words, the chief part have no notion that a word may be louder than a cannon-ball, and sharper than a sword. Cobbett is profuse of italics. This instance may be supposed to refute the assertion, that the writers who use them are not versed in the art of composition. But, although Cobbett was a wonderful master of plain speech, all his writings betray his want of logical and literary culture. He had never sacrificed to the graces; who can not be won without many sacrifices. He cared only for strength; and as his own bodily frame was of the Herculean, rather than the Apollonean cast, he thought that a man could not be very strong unless he displayed his thews. Besides, a Damascus blade would not have gashed his enemies enough for his taste; he liked to have a few notches on his sword.

**Music-Lessons.**—Porpora, one of the most illustrious composers of Italy, entertained a great feeling of friendship for a young man, a pupil of his. He asked his youthful acquaintance whether he thought he possessed courage enough to follow constantly the road he, Porpora, traced out for him, however wearisome it might appear. On receiving an affirmative reply, Porpora wrote down, upon a piece of ruled paper, the diatonic and chromatic scales, both ascending and descending, skips of thirds, fourths, fifths, etc., to teach him to master the intervals and sustain the sound, besides shakes, groups, *appogiaturi*, and other vocal exercises of various kinds. This one sheet of paper furnished both master and pupil occupation for a year; the following year also was devoted to it. The pupil began to murmur, but the master reminded him of his promise. The fourth year passed, the fifth year followed, and still there was the same eternal sheet of paper. Even during the sixth year it was not given up, though lessons in articulation, pronunciation, and declamation were added. At the end of the year, the pupil, who thought he was only engaged on the elements of his art, was surprised at hearing his master say: "There, my dear boy, you have nothing more to learn; you are the first singer in Italy." Porpora spoke the truth, for the singer was Caffarelli.

